# Enteroviruses and Type 1 Diabetes: Candidate Genes Linked With Innate Immune Response

**DOI:** 10.1016/j.ebiom.2015.06.001

**Published:** 2015-06-03

**Authors:** Enagnon K. Alidjinou, Didier Hober

**Affiliations:** Université Lille 2, Faculté de Médecine, CHRU de Lille, Laboratoire de virologie, EA3610, Lille, France; Bâtiment Paul Boulanger, Hôpital Calmette, CHRU, Boulevard du Pr Jules Leclercq, 59037 Lille, France

In addition to HLA associated genetic predisposition, the role of exogenous factors in the development of type 1 diabetes (T1D) is well admitted. Since many decades, accumulating evidence strongly has supported an association between enteroviruses (EV) and T1D (Hober and Sauter, 2010). Moreover, the potential mechanisms underlying this enteroviral pathogenesis are getting better understood, and may also be linked to background susceptibility.

Beta cell destruction/dysfunction in T1D would result from an autoimmune process (Roep and Tree, 2014) and the role of EV in the scenario should not be thought as a massive lytic replication in islets. The implication of the virus relies on the immune response, and especially the production of type 1 interferons (IFNs) and other pro-inflammatory cytokines. Indeed, the terms of the interaction between the virus and the innate immune system determine the susceptibility to this EV-mediated autoimmune diabetes, and could justify why such infection do not trigger T1D in every patient. The scenario leading to the disease is thought to include the production of significant amounts of IFNs, through activation of pathogen recognition receptors (PRRs). This inflammatory environment contributes to the initiation of autoimmune destruction of beta cells.

Candidate genes for T1D have been identified by genome-wide association studies. These genes may be involved in (i) innate immune response to infectious agents such as EV, (ii) modulation of antigen presentation and expansion of self-reactive cells or (iii) regulation of beta cell apoptosis (Concannon et al., 2009, Santin and Eizirik, 2013).

Innate immune system gene polymorphisms that are specifically related to response to viruses are very attractive to understand the early events in the enteroviral pathogenesis of T1D. This may cover molecules and pathways involved in virus recognition and IFN production as well as IFN-dependent downstream signaling and antiviral responses.

At PRR signaling level, some rare polymorphisms of IFIH1 (or MDA5) were shown to be associated to a reduced risk of T1D (Nejentsev et al., 2009). MDA5 is a well-known intracellular sensor for EV. Protective variants displaying reduced MDA5 expression are associated with a lesser response to viral infection and reduced inflammation in islets (Lincez et al., 2015).

In addition, PTPN2, a candidate gene for T1D is a negative regulator of signal transducer and activator of transcription (STAT) signaling pathway in beta cells and modulate IFN-induced beta cell apoptosis. Polymorphisms leading to a reduced expression of PTPN2 (T1D risk variant) could predispose β-cells to increased apoptosis following type 1 interferon induced by a viral infection (Colli et al., 2010).

Type 1 IFN signaling pathway also includes TYK2, another candidate gene associated with T1D (Wallace et al., 2010). Such association was already described with other autoimmune diseases like systemic lupus erythematosus or inflammatory bowel diseases. It was earlier shown that mice with natural mutation within this gene or KO models were more susceptible to infection and displayed an alteration of the response to IFNs and others pro-inflammatory cytokines. More recently this gene was clearly linked to the susceptibility of mice to a rapidly-progressing hyperglycemia induced by encephalomyocarditis virus (EMCV) (Izumi et al., 2015).

In their study, Nagafuchi et al. (2015) investigated in humans the link between TYK2 polymorphisms and the risk for diabetes. The authors identified a TYK2 promoter haplotype in patients with suspected viral infection at T1D onset. Interestingly, this variant was associated with increased risk of both type 1 and type 2 diabetes. Through its impact in the signaling of many cytokines such as types 1 & 2 IFNs, IL-6, IL-10, IL-12, IL-23, and probably others, TYK2 could be associated to inflammatory and autoimmune diseases that involve a disturbed production of these cytokines. However, due to the broad potential effect of TYK2, its specific role in the pathogenesis of virus-, and especially enterovirus-induced T1D remains an open issue.

Nagafuchi et al. (2015) claimed that the TYK2 promoter variant was associated with a more significant increased T1D risk in individuals with flu-like syndrome and in anti-GAD antibody-negative patients, but not in those with positive anti-GAD antibody. This pattern of data suggests that a TYK2 variant may be more likely associated with susceptibility to virus-induced diabetes appearing as fulminant diabetes in human beings, observed in Japan more frequently, rather than associated with autoimmune T1D.

The relationship between TYK2 and EV-related autoimmune T1D deserves further investigations. It cannot be excluded that TYK2 contributes to the pathogenesis of this disease, insofar as a strong association was reported (Wallace et al., 2010). Considering that the T1D risk allele leads to a reduced expression of TYK2 and a subsequent decreased expression of type 1 IFN-induced genes, the overall result would be a decreased antiviral response possibly involved in the mechanisms of virus persistence which can maintain an inflammatory status.

In conclusion, T1D is undoubtedly a multifactorial and polygenic disease. The interaction between environmental insults such as EV and innate immunity probably relies on a cross-talk between many genes, which confers a susceptible background.

## Disclosure

The authors declared no conflicts of interest.
